# Field efficacy of acaricides against *Varroa destructor*

**DOI:** 10.1371/journal.pone.0171633

**Published:** 2017-02-03

**Authors:** María Jesús Gracia, Carlos Moreno, Montserrat Ferrer, Alfredo Sanz, Miguel Ángel Peribáñez, Rosa Estrada

**Affiliations:** 1 Parasitología y Enfermedades Parasitarias, Departamento de Patología Animal, Facultad de Veterinaria, Universidad de Zaragoza-CITA, Zaragoza, Spain; 2 Genética Animal, Departamento de Anatomía, Embriología y Genética Animal, Facultad de Veterinaria, Universidad de Zaragoza-CITA, Zaragoza, Spain; 3 Servicio de Ordenación y Sanidad Animal, Departamento de Agricultura y Alimentación, Diputación General de Aragón, Zaragoza, Spain; 4 ARNA, Asociación Apícola, Zaragoza, Spain; University of Cologne, GERMANY

## Abstract

Field trials were conducted in Northeast Spain (Aragón) to evaluate the effectiveness of two acaricides against *Varroa destructor*. These experiments took into account the season of the year, apiary, colony, and developmental state and strength of the colony. The acaricides used were a synthetic (amitraz, Apivar®) and a natural (formulated from Api Life Var®, thymol oil and thymol alcohol) product. The treatments used in the present study reduce high infestations of *V*. *destructor*, although they do not eliminate the infestation. Similar efficacies between treatments were found. Nevertheless, the efficacy of a treatment depends on the apiary where applied. Moreover, the detected variability in the apiary and hive poses a challenge to the identification of the significant factors. Therefore, more field studies to assess efficacies in several apiaries are needed to obtain a better understanding of the effects of the applied treatments.

## Introduction

*Varroa destructor (Anderson and Trueman) (Acari*: *Mesostigmata)* is the main parasite of *Apis mellifera*, and it can cause the collapse of untreated colonies in a few years. Mite control is imperative to maintain the honey bee colonies in most beekeeping regions around the world. Synthetic acaricides such as fluvalinate, flumetrine, amitraz, coumaphos, and cymiazole have been used successfully to control *V*. *destructor*. However, pesticides can leave residues in the wax and honey, and in recent years, the intensive utilization of many chemicals against *V*. *destructor* has resulted in the development of resistance in the mites to acaricides [1].

For these reasons, alternative methods of control have been promoted. Over the past few years, the worldwide trend has been to use natural substances, particularly some organic acids, especially formic acid [2] and oxalic acid [3], and essential oils and their components, especially thymol [4–14]. The general advantages of these natural compounds are sufficient efficacy against *V*. *destructor* and the low risk of residues and accumulation in bee products [11]. A major criticism of using plant-derived treatments for *Varroa* control is that their efficacy is less reliable [1].

Amitraz and thymol are two of the main products used to control *V*. *destructor* in Spain, both having acaricidal activity, whereas thymol also has repellent effects [15]. Amitraz (a formamidine pesticide) acts on the target pest species by interacting with the octopamine receptor of the central nervous system and is known as a neurotoxic, sublethal miticide [16]. Amitraz is spread in the colony from the contact between the honey bees and the plastic strips containing the active ingredient. Its effectiveness has been demonstrated with values from 83.8% to 99.5% [17–21]. Thymol is a constituent of plant-derived essential oils. The miticidal action of thymol is exerted mainly by evaporation on a support but also by contact, as bees access the medium, and thereby "disintegrate" and "disperse it" throughout the hive. Its effectiveness has been demonstrated with values ranging from 70% to 97% [4–14].

Numerous factors contribute to the overall efficacy of an acaricide, including the concentration of the compound involved, treatment period, and the colony and apiary environment. Moreover, the efficacy of some compounds depends on the evaporation pressure within the colony; therefore, the time of year or the ambient temperature during treatment application [6] can influence the effectiveness of the treatment. Bee activity influences the amount of the active substance that will be distributed on the surface of the bees [21]; therefore, a larger population could favor the dispersion of the product and consequently achieve greater efficacy [10, 14]. Other factors, such as the amount of brood in the hive [21, 22], the severity of the infestation [10, 23], delivery method used [10, 11, 15, 24], and hive type [6], have also been suggested to affect treatment efficacy. Because different factors may influence the effectiveness of an acaricide, field studies to establish this possibility should be carried out in different apiaries [25].

Efficient control of the honey bee parasite *V*. *destructor* is a major concern for beekeepers around the world. For this reason, the present study intends to evaluate, in different apiaries, the effectiveness of a synthetic (amitraz, Apivar®) and a natural product (Api Life Var®, thymol oil and thymol alcohol) against *Varroa destructor* in relation to the season of the year, apiary, colony, and the developmental state and strength of the colony.

## Materials and methods

The study was carried out in Northeast Spain (mid-Ebro valley, Aragón), in eight apiaries of 25 colonies each. The climate in the Ebro River valley is Mediterranean continental, with little rain (average annual rainfall 300–400 mm). Most precipitation falls in autumn and spring, while winter and summer are dry. The climate is characterized by important diurnal and seasonal oscillations. The average median temperature is 25°C in July and August (absolute maximum above 40°C) and the coldest month is January with an average of 5°C (absolute minimum below -10°C).

The treatments were applied during a period of 6 weeks in autumn (September-November) and spring (April-June) (four apiaries each season) outside the honey flow, when honey bees are active and a sealed brood is usually present. The apiaries were composed of Langstroth hives. All hives were occupied by *A*. *mellifera* colonies naturally infested by *V*. *destructor*. The honey bee colonies had not received any treatment for a year before the field trials. The study was carried out on private land, and the landowners gave permission to conduct the study on these sites. No specific permissions were required for these locations/activities, and the field studies did not involve endangered or protected species. All relevant regulations (e.g. national, legal) were followed when applying the pesticide mixtures and conducting the experiments.

In each apiary, the hives were randomly arranged into five groups, A, B, C, D and E, of five hives each (Table 1). Colonies of group A each received Api Life Var®, (Chemicals LAIF, Vigonza, Italy); colonies of group B each received Apivar® (Laboratorios Calier S.A. Barcelona, Spain); colonies of group C each received a mix of thymol in olive oil; colonies of group D each received a mix of thymol in ethanol; and colonies of group E were used as control and received no treatment. The two thymol homemade preparations, thymol oil and thymol alcohol, were produced following the methodology of previous assays [8, 10] by mixing thymol (PRS Panreac minimum purity of 99–101%) in oil or ethanol, respectively. The oil or ethanol (20 ml) was heated, and the thymol (15 g) was added during agitation until complete dissolution was achieved. The mix was poured into a piece of floral foam, which was sealed to prevent evaporation prior to its use in the apiary.

**Table 1:** Description of the experimental groups.

Group	A	B	C	D	E
**Name**	Api Life Var®	Apivar®	Thymol-oil	Thymol-alcohol	Control
**Active ingredient**	thymol, eucalyptol, menthol, camphor	amitraz	Thymol	Thymol	No a.i.
**How applied**	Vermiculite carrier matrix	Polyethylene strips	Floral foam (8x5x1 cm)	Floral foam (8x5x1 cm)	
**Dose**	2 tablets x 10 g	2 strips x 0.5 g	2 floral foam x 15 g	2 floral foam x 15 g	
**Localization**	Upper part of the brood combs	Into the brood nest	Upper part of the brood combs	Upper part of the brood combs	
Duration*	42 days (replace at 21 days)	42 days	42 days (replace at 21 days)	42 days (replace at 21 days)	

* The duration and repetition of treatments follow the maker´s guidelines.

Before and after each treatment, the colony strength and the mite infestation levels were monitored. Colony strength indicates the potential of a hive of bees to pollinate horticultural or agricultural crops and is evaluated by estimating the number of adult honey bees and the amount of brood in a hive. For that, the surface occupied by adult and immature worker honey bees (eggs, larva and capped brood) on each side of every frame (frame size: 42 x 20 cm) was evaluated [21, 26, 27] and the number of frames occupied by adult and brood allows us to quantify the colony strength. For each colony, the infestation rates in the adult bees and brood were measured on day 0 (initial infestation) and on day 42 (final infestation) of the treatment. For the adult bees, approximately 500 workers from each colony were obtained by brushing one comb taken from the center of the brood nest. They were placed in a plastic pot containing ethanol (25%, in water) which was shaken vigorously. The mites were separated from bees by means of a 3 mm sieve placed on top of a 0.1 mm sieve [28, 29]. The mites and bees were counted and the results were expressed as the percentage of the ratio of number of mites/number of bees. For the sealed brood, approximately 400 cells of the sealed brood- each one contained one worker pupa—were examined per colony. To obtain a representative sampling; the samples were taken from the center of the brood nest from a minimum of three different frames (near, mid and far of treatment). The infestation was measured by a standardized method [29, 30]. Briefly explained, it consisted on opening individual cells, extracting larva, pre-pupa or pupa and examining cell walls using an appropriate source of light to finally confirm the presence of the mites themselves in the cell or on the brood. The results were expressed as the percentage of the number of mites/number of brood cells. Table 2 shows the initial strength and the initial (final) infestation in adult and brood for each treatment in each apiary.

**Table 2:** Strength, expressed as mean number of initial frames (MNF), mean initial (MII) and mean final infestation (MFI) of the raw data, the last two concepts expressed as a percentage, for each combination of apiary, physiological stage and treatment levels. Blank boxes: no brood in the colony.

		Api Life Var	Apivar	Thymol oil	Thymol alcohol	Control
**Apiary 1**						
**Adult**						
	**MNF**	6.3	5.3	6.0	6.8	4.5
	**MII**	7.6	26.4	11.5	10.8	11.5
	**MFI**	0.3	0.4	0.1	2.4	15.6
**Brood**						
	**MNF**	4.7	3.5	5.3	6.0	4.0
	**MII**	2.9	12.6	8.0	2.1	23.4
	**MFI**	0.3	0.6	0.0	7.2	47.2
**Apiary 2**						
**Adult**						
	**MNF**	16.0	16.0	13.8	13.7	13.2
	**MII**	1.3	3.5	6.8	9.6	8.3
	**MFI**	0.2	0.8	1.2	1.5	15.3
**Brood**						
	**MNF**	7.3	6.3	8.2	4.8	7.0
	**MII**	6.9	22.2	17.8	36.0	23.9
	**MFI**	0.0	24.1	0.3	6.9	67.1
**Apiary 3**						
**Adult**						
	**MNF**	7.6	8.0	7.6	7.4	7.0
	**MII**	9.2	12.4	13.3	11.7	13.9
	**MFI**	2.8	0.7	0.7	8.6	26.1
**Brood**						
	**MNF**	6.0	6.7	6.4	6.2	4.8
	**MII**	17.0	8.2	14.0	11.6	23.5
	**MFI**	9.2	3.8	1.2	11.3	42.9
**Apiary 4**						
**Adult**						
	**MNF**	8.4	9.6	9.8	5.2	5.8
	**MII**	14.9	16.5	6.4	8.8	5.4
	**MFI**	1.6	0.6	0.9	0.3	10.4
**Brood**						
	**MNF**	6.0	5.8	6.0	2.0	4.3
	**MII**	8.8	35.6	11.1	23.8	6.5
	**MFI**	9.0	3.3	3.4	0.0	16.8
**Apiary 5**						
**Adult**						
	**MNF**	13.8	18.8	14.3	7.0	17.0
	**MII**	8.9	8.1	2.1	0.7	5.2
	**MFI**	1.9	0.3	1.2	0.0	5.6
**Brood**						
	**MNF**	9.0	5.7	8.0	3.3	5.5
	**MII**	27.2	85.0	4.6	5.3	6.1
	**MFI**	1.8	7.4	7.0	0.0	21.3
**Apiary 6**						
**Adult**						
	**MNF**	6.4	6.0	6.8	6.4	8.8
	**MII**	7.5	9.5	14.0	11.4	14.7
	**MFI**	0.0	0.0	1.1	1.2	23.0
**Brood**						
	**MNF**		2.0	3.0	3.5	3.0
	**MII**		40.7	118.2	50.9	39.0
	**MFI**		0.0	2.4	0.0	35.8
**Apiary 7**						
**Adult**						
	**MNF**	9.4	8.7	10.8	8.8	9.4
	**MII**	13.5	1.4	2.6	1.1	1.7
	**MFI**	1.3	2.8	1.2	1.1	6.1
**Brood**						
	**MNF**	2.5		2.5	2.0	2.5
	**MII**	24.2		46.7	1.1	5.7
	**MFI**	4.9		4.9	0.0	12.5
**Apiary 8**						
**Adult**						
	**MNF**	7.2	10.4	7.8	7.6	9.2
	**MII**	6.3	7.4	5.9	8.4	12.4
	**MFI**	2.5	0.1	2.7	2.2	13.3
**Brood**						
	**MNF**					
	**MII**					
	**MFI**					

### Statistical analysis

In this experiment, efficacy is the studied trait. Specifically, efficacy of the “i_th_” hive for a concrete stage (adult or brood) is defined as the ratio between the final infestation and initial infestation with respect to the mean of the same ratio obtained from the control hives of the apiary where the hive is placed:

$${Efficacy}_{i}=\left\{({FI}_{i}/{II}_{i})/\sum_{j=1,n_{c}}({FI}_{i}/{II}_{i})/n_{c}\right\}$$

where FI_i_ (II_i_) is the final (initial) infestation of the “i_th_” hive for a concrete stage (adult or brood), FI_j_ (II_j_) is the final (initial) infestation of the “j_th_” control hive for a concrete stage, and n_c_ is the number of control hives of the apiary where the “i_th_” hive is placed. Moreover, the final (initial) infestation is expressed as the percentage of mites in adult or brood stages in the “i_th_” hive after (before) the treatment.

Because the definition of efficacy is a ratio between the final and initial infestation, the most efficient treatment is that with a zero value of efficacy. Moreover, with this definition, the final infestation is related not only to the initial level of infestation but also to the development of the infestation process in the corresponding apiary. In this sense, to reduce the initial infestation in an apiary where the infestation in control hives has increased does not show the same efficacy as when the infestation has decreased.

Efficacy is justified with the following mixed linear model:

$$Y_{ijklm}=\mu +s_{i}+a_{ij}+t_{k}+c_{ijkl}+p_{m}+b(x_{ijklm}-\mu_{x})+s_{i}t_{k}+s_{i}p_{m}+t_{k}a_{ij}+t_{k}p_{m}+p_{m}a_{ij}+s_{i}t_{k}p_{m}+a_{ij}t_{k}p_{m}+e_{ijklm},$$

where Y_ijklm_ is the efficacy obtained in the “l_th_” hive located in the “j_th_” apiary of the “i_th_” season when the “k_th_” treatment is applied in the “m_th_” stage; μ is the population mean; s_i_ is the season effect (spring (s_1_), autumn (s_2_)); a_ij_ is the apiary effect, with four apiaries analyzed in spring and the other four in autumn; t_k_ is the treatment effect, also with four levels (Api Life Var® (t_1_), Apivar® (t_2_), thymol-oil (t_3_) and thymol-alcohol (t_4_)); c_ijkl_ is the hive effect, with a maximum of five hives for each combination of apiary and treatment; p_m_ is the physiological stage effect (adult (p_1_), brood (p_2_)); b is the coefficient of regression defined between efficacy and the initial number of frames in the corresponding hive (x_ijklm_), being μ_x_ the mean number of frames in the population; s_i_t_k_ is the season-treatment interaction; s_i_p_m_ is the season-physiological stage interaction; t_k_a_ij_ is the treatment-apiary interaction; t_k_p_m_ is the treatment- physiological stage interaction; p_m_a_ij_ is the physiological stage-apiary interaction; s_i_t_k_p_m_ is the season-treatment-physiological stage interaction; a_ij_t_k_p_m_ is the apiary-treatment-physiological stage interaction; and e_ijklm_ is the residual term.

In this model, all factors were assumed to be fixed, except apiary, hive and the residual, which are random. The proposed model is solved using the Univariate General Linear Model option of the IBM SPSS Statistics program. Furthermore, a backward stepwise selection strategy has been followed to reach the final model, where the factor with a greater p-value is eliminated in each step. This process stops when only factors with a p-value lower or equal to 0.2 or factors that are essential for the architecture of the model remain.

## Results

The mean initial and final infestation, and mean efficacy of the raw data are shown in Table 3 for each combination of the season-physiological stage-treatment levels. The results of this Table lead us to suspect that the season-treatment-physiological stage interaction can be important (in spring, the efficiency mean of the thymol alcohol in the brood is worse than in the adult, whereas in autumn, it is just the opposite).

**Table 3:** Number of observations (N), mean number of initial frames (MNF), mean initial infestation (MII), mean final infestation (MFI), and mean efficacy of the raw data (ME, standard error is in parentheses), expressed as a percentage, for each combination of season, physiological stage and treatment levels. In the right margin, the estimated marginal mean efficacy (standard error is in parentheses) for each treatment is shown.

	Spring		Autumn		
	Adult	Brood	Adult	Brood	
**Api Life Var**					
**N**	18	15	20	8	
**MNF**	9.9	6.1	9.2	5.8	
**MII**	8.3	9.8	9.0	25.7	
**MFI**	1.3	4.9	1.4	3.3	
**ME**	9.5(2.7)	19.8(9.8)	18.0(5.4)	9.1(6.4)	12.1(3.2)
**Apivar**					
**N**	19	15	17	4	
**MNF**	10.0	5.5	10.8	4.8	
**MII**	14.1	20.4	7.1	73.9	
**MFI**	0.6	8.2	0.6	5.5	
**ME**	8.2(3.1)	20.2(7.6)	4.3(2.5)	18.3(16.8)	11.7(3.4)
**Thymol oil**					
**N**	19	19	17	5	
**MNF**	9.5	6.5	9.4	4.8	
**MII**	9.4	13.0	6.9	44.2	
**MFI**	0.8	1.3	1.6	5.2	
**ME**	13.8(6.5)	5.0(3.3)	23.7(8.9)	17.8(16.2)	11.6(3.1)
**Thymol alcohol**					
**N**	18	13	16	6	
**MNF**	8.1	5.1	7.4	3.2	
**MII**	10.2	19.5	6.5	19.8	
**MFI**	3.3	8.8	1.3	0.0	
**ME**	10.2(3.3)	52.9(24.7)	16.1(5.7)	0.0(0.0)	17.5(3.5)

**Fig 1:**
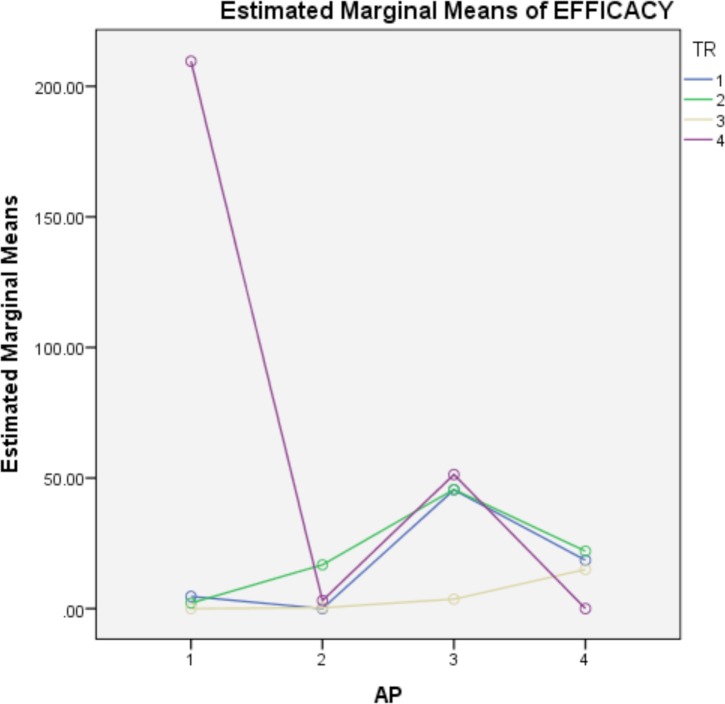
Interaction between the apiaries of spring and the treatments on the brood.

The results of the analysis of variance applied to the data are displayed in Table 4, where only the covariate has been eliminated because of its lack of signification. Hence, in the obtained final model, the season-treatment-physiological stage interaction is very close to significance, whereas the apiary-treatment-physiological stage interaction is clearly significant, as are the physiological stage-apiary and the treatment-apiary interactions, and the apiary factor. To understand the significance of the apiary-treatment-physiological stage interaction, Fig 1 shows the interaction between the first four apiaries (apiaries of spring) and treatments at the second level of stage (brood), with this being the only conditional interaction that is significant. The same interaction at the first level of stage (adult) is not significant, as it happens, as well as the interaction between the apiaries of autumn and treatments at both levels of stage. Clearly, the significance of this conditional interaction is justified by the poor effect of treatment four (thymol-alcohol) in the first apiary. In fact, when an analysis of the residuals is conducted, an outlier is detected, which corresponds to the efficacy of this treatment obtained in the brood of the third hive. The value of this observation (293.4%) suggests an error, justifying its exclusion from the analysis.

**Table 4:** Results of the analysis of variance corresponding to efficacy.

Source	Degrees of freedom	Mean square	F	p-value
**Season**	1	1598.4	0.550	0.4863
**Apiary**	6	2906.2	4.181	0.0008
**Treatment**	3	1086.2	0.379	0.7693
**Hive**	114	695.2	1.338	0.1120
**Phys. stage**	1	3435.5	1.156	0.3314
**Season-Treat. inter.**	3	250.6	0.087	0.9663
**Season-Phys. inter.**	1	1831.0	0.616	0.4681
**Treat.-Apia. inter.**	18	2864.6	4.121	0.0000
**Treat.-Phys. inter.**	3	2506.8	1.098	0.3850
**Phys.-Apia. inter.**	5	2972.6	5.721	0.0002
**Seas-Treat-Phy inter.**	3	1380.8	2.657	0.0569
**Api-Treat-Phy inter.**	13	2282.8	4.393	0.0000
**Residual**	57	519.6		

When an analysis of variance is done from the data without the outlier, the results shown in Table 5 are obtained where, because of the backward stepwise selection strategy applied, the covariate, the season-treatment-physiological stage interaction and the apiary-treatment-physiological stage interaction are eliminated from the model. Moreover, the Akaike’s Information Criterion of this final model is 857.4, whereas the value of this criterion in the complete model is 945.2; this result, therefore, confirms the adequacy of the achieved model. Hence, as presented in Table 5, the significant factors of the model are the apiary, the hive, and the treatment-apiary and the physiological stage-apiary interactions, whereas the treatment-physiological stage interaction is very close to significance.

**Table 5:** Results of the analysis of variance corresponding to efficacy from the data without the outlier.

Source	Degrees of freedom	Mean square	F	p-value
**Season**	1	5302.3	2.491	0.1656
**Apiary**	6	2128.8	3.255	0.0054
**Treatment**	3	353.8	0.314	0.8150
**Hive**	114	654.1	1.461	0.0420
**Phys. stage**	1	585.5	0.507	0.5083
**Season-Treat. inter.**	3	430.2	0.382	0.7670
**Season-Phys. inter.**	1	338.8	0.293	0.6111
**Treat.-Apia. inter.**	18	1125.6	1.721	0.0452
**Treat.-Phys. inter.**	3	1182.1	2.641	0.0561
**Phys.-Apia. inter.**	5	1154.9	2.580	0.0332
**Residual**	72	447.7		

**Fig 2:**
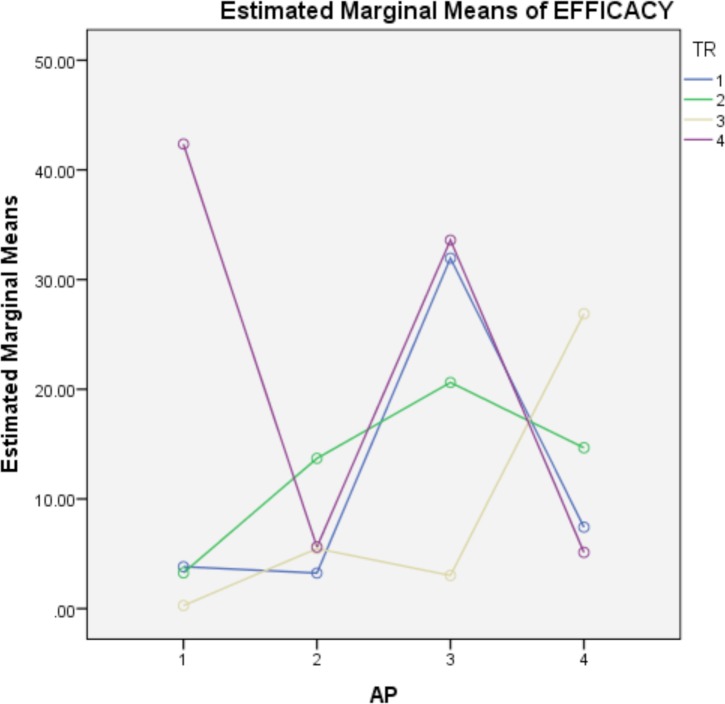
Interaction between the apiaries in spring and the treatments.

To explain the significant treatment-apiary interaction, Fig 2 shows the interaction between the first four apiaries (spring apiaries) and the treatments, which is the only partial interaction that is significant (the interaction between the apiaries in autumn and the treatments is not significant). In this Fig, the treatment effect is clearly shown to depend on the apiary where applied, as observed with treatment four (thymol alcohol), which shows the least efficacy in the first apiary and the best in the fourth.

**Fig 3:**
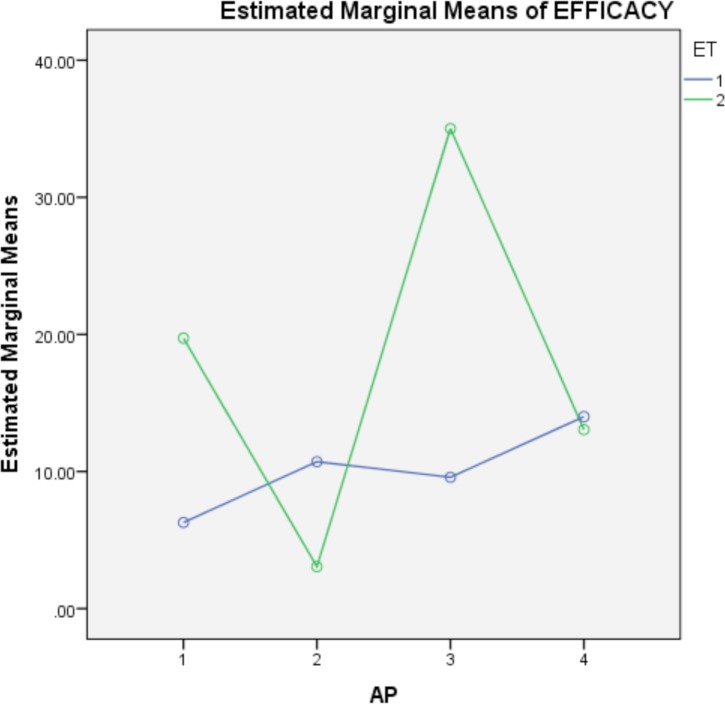
Interaction between the apiaries in spring and the physiological stage.

With respect to the physiological stage-apiary interaction, Fig 3 shows the only partial significant interaction between the apiaries in spring and the physiological stage. This Fig indicates that the efficacy in the adult or brood stages depends on the location of the apiary. In this sense, the efficacy registered in the brood in the second apiary is the best, whereas it is the worst in the third one.

**Fig 4:**
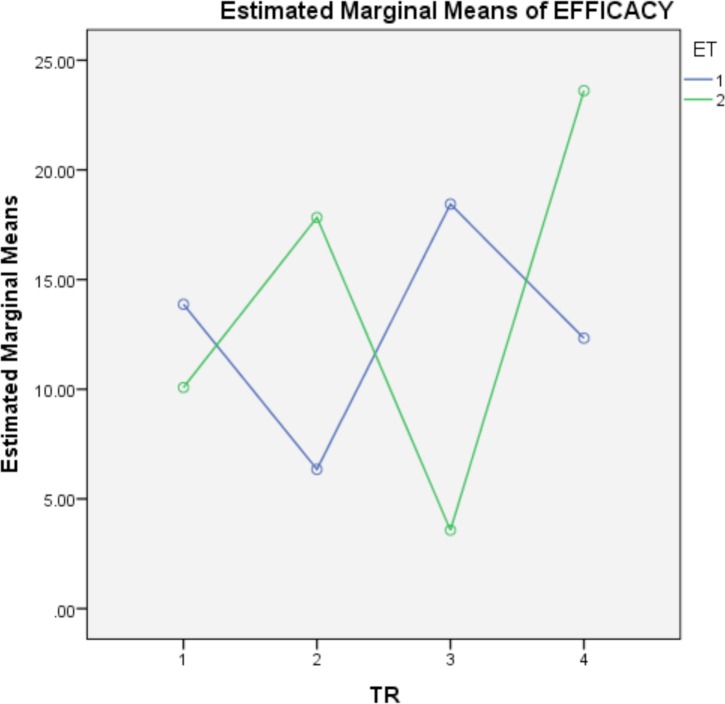
Interaction between treatments and physiological stage.

Finally, although the treatment-physiological stage interaction is not significant, the analysis of this interaction was interesting. Fig 4 shows that the efficacy of treatments could depend of the stage when applied.

## Discussion

As shown in the right margin of Table 3, all treatments decrease the level of infestation of *V*. *destructor* although they do not eliminate the infestation. In our work, the similarity in efficacy among the four treatments was notable. We might have expected greater efficacy from amitraz (Apivar®), a synthetic acaricide in comparison with the natural products [1]. Nevertheless, no significant differences were observed between them, whether the product was marketed or handmade. We think that these results cannot be justified as a consequence of the repetition of some but not all treatments (one application of Apivar® compared to other treatments which must be replaced after twenty-one days) because the treatments were applied following the manufacturer's recommendations. The formulation of the product appeared to be one of the major factors affecting its effectiveness [10, 11, 15, 24], and important differences were observed between the different formulations of thymol [11]. In our work, the lack of statistical significance in the efficacy between a marketed product, Api Life Var®, and the handmade products, thymol dissolved in oil and thymol dissolved in alcohol was interesting. Anyway, although there are no differences between the two kinds of products (marketed *vs* handmade treatments) the use of homemade chemical mixtures in honeybee colonies to control *V*. *destructor* should follow the specific legal regulations of each country. Nevertheless, as discussed below, the most important result obtained from the treatments was the different effect of the treatment as a function of the apiary where applied.

Spring and autumn constitute the most common seasons for beekeepers to apply control measures for *Varroa*, and thus any difference between how the treatments work in spring or autumn is important. This is especially important for those treatments most dependent on external temperature [31], as with the products that evaporate (thymol) compared to those spread by contact (amitraz). Manufacturers recommend treatment in spring and/or autumn; however, application during the late autumn is usually not effective enough, and studies show that in autumn the parasitism in the brood is higher and could cause a decreased effectiveness of the acaricides [10, 23, 32]. However, with respect to season, in our trial, no differences were detected between the seasons. Moreover, the lack of significance of the season-treatment interaction means that we cannot recommend one treatment over another with regard to the time of application.

In this experiment, a statistical significance of efficacy between the apiaries was detected, a result that agrees with those previously obtained [21, 25]. Moreover, the interaction between treatment and apiary is also significant, which states that the efficacy of the treatment depends on the apiary where is applied, particularly when treatments are carried out in spring. This is especially evident (Fig 2) with treatment four (thymol alcohol), which showed the worst efficacy in the first apiary and showed the best in the fourth one. Other interesting responses are shown by the good efficacy of Api Life Var® (treatment 1) in apiaries one, two and four against the poor results in apiary three; or by the different efficacy of treatment three (thymol oil) in apiary one *versus* the results in apiary four. The efficacy depends on the apiary to which treatment is applied but we can see a similar efficacy in some treatments. In spring, except apiary 1, the efficacy of treatment 1 (Api Life Var®) is identical to that of treatment 4 (thymol alcohol); good efficacy in apiaries 2 and 4 and poor efficacy in apiary 3. This could be due to the use of the same active ingredient and that the homemade preparation of thymol alcohol acts in a manner more similar to the commercial product Api Life Var® than the other treatment with thymol (thymol oil). Although not as obvious, a similar efficacy is also observed for treatments 2 and 3 (Apivar® and thymol oil), depending on the apiary where the test is performed. One possible explanation for this similarity of two products with different a.i. and way they spread in the colony is that the oil, in certain circumstances, does not allow the evaporation of thymol and acts more like a contact acaricide. That is, certain conditions occurring in apiaries cause similarity in effectiveness of acaricides. Most trials of efficacy against *Varroa* have been performed in a single apiary, but the management, variety of bees used, humidity, temperature and other location-dependent factors influence the results; therefore, testing in a single apiary can yield inaccurate results. Our study confirms the need to design experiments where treatments are proven in various apiaries.

The statistically significant differences found among the hives are interesting. This result agrees with reports from other authors; significant variability in the level of susceptibility to amitraz between the mite populations originating from different colonies in the same apiary has been noted [33]. In addition, a high colony-to-colony variability of the thymol effectiveness against *V*. *destructor* was found [6, 11, 24], suggesting that the high variability in the efficacy of the volatile compound was influenced by biological and/or climatic factors [11] and the model of the hive [6]. Our work does not allow us to draw explicit conclusions on the causes of the different efficacy of treatments among the hives. The active substance dosage acting on the *Varroa* mites through the adult bees is of decisive importance; thus, not only the amount of active ingredient contained in the treatment but also the bees’ activities influence the amount of the active substance that will appear on the surface of the bees’ bodies [21]. The efficacy of an acaricide can be conditional on the colony environment and especially on its placement in the apiary. That is, a hive placed in a sunny and warmer area would allow a more effective treatment; the external temperature not only would affect the release of the treatment but would have a bigger impact on the activity of the honey bee colonies.

With regard to the developmental state, we might think that the treatments are more effective against the phoretic mites on the workers than in the brood population, especially if we consider the life cycle of this parasite and the shelter the mite receives in the capped cells [1]. However, in this assay, an absence of differences in the efficacy of treatments relative to the developmental state was found. Nevertheless, the significant physiological stage-apiary interaction must be highlighted. Our results indicate that the efficacy in the adult or brood state in the spring depends on the apiary where the treatments are applied (Fig 3). In this sense, we obtain the best efficacy in the brood in apiary two and the worst in the apiary three, and similar efficacies in apiaries one and four, whereas the efficacy registered in the adult is similar in the four apiaries. Another interesting point is that, although the treatment-physiological stage interaction is not significant, Fig 4 indicates that the efficacy of the treatments could depend of the stage where applied. Hence, according to the conditions under which this study was conducted, we could recommend one treatment or another depending of the size of the brood present in the hive; when a small brood is present, we would recommend Apivar®, whereas in times of increased brood, we recommend the use of thymol oil.

Finally, it is also important to know which treatments will work best depending on the strength of the hive. A larger population could favor the dispersion of the product and consequently would achieve greater efficacy [10, 14]. In the present study, the lack of significance of the covariate introduced in the model (the number of initial frames of the hive) indicates that no differences in efficacy were found relative to this aspect. Hence, we cannot recommend a concrete treatment according to the strength of the hive.

## Conclusions

Similar efficacies between treatments were found. Nevertheless, the efficacy of the treatment depends on the apiary where applied. Moreover, the detected variability of the apiary and hive poses a challenge regarding the identification of those factors that are significant. Therefore, more field studies are necessary to assess the efficacies in several apiaries and thereby obtain a better understanding of the effects of the applied treatments.
